# Comparative analysis of methods for identifying multimorbidity patterns among people with opioid use disorder: A retrospective single-cohort study

**DOI:** 10.1371/journal.pone.0324548

**Published:** 2025-06-12

**Authors:** Myanca Rodrigues, Tea Rosic, Glenda Babe, Brittany B. Dennis, Alannah McEvoy, Richard Perez, Claire de Oliveira, Sameer Parpia, Zainab Samaan, Lehana Thabane

**Affiliations:** 1 Department of Health Research Methods, Evidence, and Impact, McMaster University, Hamilton, Ontario, Canada; 2 Department of Psychiatry, University of Ottawa, Ottawa, Ontario, Canada; 3 Children’s Hospital of Eastern Ontario Research Institute, Ottawa, Ontario, Canada; 4 The Centre for Addiction and Mental Health, Toronto, Ontario, Canada; 5 British Columbia Centre on Substance Use, Vancouver, British Columbia, Canada; 6 Division of Social Medicine, Department of Medicine, University of British Columbia, Vancouver, British Columbia, Canada; 7 Department of Medicine, McMaster University, Hamilton, Ontario, Canada; 8 Department of Psychiatry and Behavioural Neurosciences, St. Joseph’s Healthcare Hamilton, Hamilton, Ontario, Canada; 9 ICES, Toronto, Ontario, Canada; 10 Institute of Health Policy, Management and Evaluation, University of Toronto, Toronto, Ontario, Canada; 11 Department of Oncology, McMaster University, Hamilton, Ontario, Canada; 12 Mood Disorders Program, St. Joseph’s Healthcare Hamilton, Hamilton, Ontario, Canada; 13 Department of Psychiatry, Queen’s University, Kingston, Ontario, Canada; 14 Biostatistics Unit, Research Institute at St Joseph’s Healthcare, Hamilton, Ontario, Canada; 15 Departments of Pediatrics/Anesthesia, McMaster University, Hamilton, Ontario, Canada; 16 Faculty of Health Sciences, University of Johannesburg, Johannesburg, South Africa; Lahore College for Women University, PAKISTAN

## Abstract

**Background:**

Multimorbidity, the presence of two or more (2+) chronic conditions, presents significant challenges for healthcare delivery, particularly among populations with opioid use disorder (OUD). Multimorbidity patterns among individuals with OUD are not well established, and minimal research exists examining the impact of clustering methods on identifying these patterns.

**Objective:**

Our study aimed to assess multimorbidity prevalence, explore associated sociodemographic and clinical characteristics, and determine multimorbidity patterns using hierarchical cluster analysis (HCA) and K-means clustering among people receiving treatment for OUD in Ontario, Canada between 2011 and 2021.

**Methods:**

Data from two prospective cohort studies were merged and linked to Ontario provincial health administrative databases. We identified 16 chronic conditions, used in prior research examining multimorbidity in Ontario, using ICD-10-CA diagnostic codes and the diagnostic codes of physician billing claims using a 2-year lookback. Multimorbidity was defined as the presence of 2+ of the above conditions, excluding the diagnosis of OUD. We conducted a retrospective cohort study, following the participants for eight years in the data holdings to ascertain the prevalence of multimorbidity. Sociodemographic and clinical characteristics were analyzed using modified Poisson regression models, and multimorbidity patterns were identified through HCA and K-means clustering.

**Results:**

Among 3,430 people with OUD, 32.5% (*n* = 1,114, 95% confidence interval (CI)=30.9, 34.1) experienced multimorbidity over an eight-year period, with older age (Prevalence Ratio (PR)=3.39, 95% CI = 2.36, 4.87) and unemployment (PR = 1.31, 95% CI = 1.13, 1.54) associated with increased prevalence. HCA identified six distinct disease clusters, whereas K-means clustering identified four clusters. Both methods identified groupings of cardiovascular (coronary syndrome), cardiometabolic (diabetes, hypertension), and respiratory (chronic obstructive pulmonary disease) diseases, reflecting shared comorbidities among people with OUD.

**Discussion:**

Our findings highlight the substantial burden of multimorbidity among populations with OUD, and the importance of considering sociodemographic factors in understanding multimorbidity prevalence. Moreover, the choice of clustering method significantly influences the identification and interpretation of multimorbidity patterns, with HCA providing more clinically meaningful groupings compared to K-means clustering. Our findings highlight the need for clinicians to tailor care plans and for policymakers to prioritize integrated healthcare delivery strategies to address the complex health needs of people with OUD.

## Introduction

Opioid use disorder (OUD) is a pubic health crisis in North America with 21 daily deaths and 15 hospitalizations in Canada as a result of opioid-related poisoning [1,2]. An estimated 351,000 people currently living with OUD nationwide [3]. Similarly, the number of opioid-related fatalities increased from 49,860 in 2019–81,806 in 2022 in the United States [4]. Prior research has demonstrated that several chronic diseases are highly prevalent among people with OUD, including cancer, chronic obstructive pulmonary disease (COPD), depression, diabetes, hypertension, and kidney dysfunction [5–9]. The presence of co-occurring chronic diseases can lead to direct challenges for health providers in the management of OUD, as some conditions may result in deleterious consequences for health and other substance use outcomes. For instance, patients with substance use disorders who additionally present with certain comorbidities, e.g., congestive heart failure (CHF), experienced a higher rate of patient-initiated discharge, in turn leading to worse health outcomes, including hospital readmission [10]. While previous research has highlighted the prevalence of individual chronic diseases among this clinical population, the concept of *multimorbidity* – the co-occurrence of two or more (2+) co-occurring chronic conditions [11], is now an emerging topic in the literature and often seen in clinical practice [12].

Studies in other jurisdictions have demonstrated a six-fold increase in the incidence of multimorbidity among people with OUD currently engaged in opioid agonist treatment (OAT), as compared to matched controls (odds ratio = 6.6, 95% confidence interval (CI)=4.3, 10.2) [13]. Furthermore, prior research has identified distinct patterns of multiple co-occurring chronic disease among people with OUD, including: psychiatric disorders, cancer, and a clustering of diabetes and renal disease [14]. However, there is a paucity of evidence on the prevalence and patterns of multimorbidity among people with OUD on OAT in Canada, with no Canadian study conducted to date.

The prevalence of multimorbidity is commonly assessed by physician billings and/or hospitalisation records in health administrative data [15,16], while systematic reviews have identified cluster analysis and exploratory factor analysis (EFA) as among the most commonly-used methods to assess patterns of co-occurring chronic diseases [17–19]. A recent comparison of these techniques has demonstrated cluster analysis as more useful than EFA for an in-depth study of multimorbidity patterns [20]. In the context of multimorbidity, cluster analysis identifies patterns of co-occurring chronic conditions by examining differences between disease. Clusters typically comprise diagnoses that share similarities (measured by Euclidean distances), with each diagnosis assigned to only one cluster [21]. Two main forms of cluster analysis include hierarchical cluster analysis (HCA) and non-hierarchical cluster analysis (NHCA), with the K-means method being the most common NHCA technique [21]. HCA merges or splits entities to form clusters, aiming to create relatively homogeneous groups of diseases. These clusters consist of diagnoses sharing common characteristics, with each diagnosis assigned to a single cluster [21,22]. Alternatively, NHCA techniques directly assign patients to clusters once the desired number of clusters is specified, without iterative division or clustering of data points. While HCA has primarily been used to identify multimorbidity patterns [17–19], it is susceptible to outliers and may be inefficient in analyzing large datasets, raising concerns about the robustness of findings [21]. NHCA, in particular K-means, has been demonstrated to be less sensitive to outliers and efficient for analyzing large datasets in population-based studies of multimorbidity patterns [21,23–26].

Understanding multimorbidity patterns among people living with OUD is critical for developing tailored treatment approaches, which address the complex health needs of this population, particularly in the Canadian setting, where there is currently limited evidence. As the number of complex comorbid conditions rises, the management of the OUD population becomes increasingly challenging, with direct implications on health and substance use outcomes [27]. By identifying common clusters of chronic conditions through multiple methodological techniques such as HCA and NHCA, clinicians can better anticipate and manage potential interactions between medications used to treat OUD and those prescribed for concomitant diseases [28,29]. This comprehensive approach enhances the robustness of findings and provides a more comprehensive understanding of the burden of disease experienced by people with OUD, ultimately aiming to improve health outcomes for this clinical population [30].

For these reasons, our study aims to:

1. Assess the prevalence of multimorbidity among people treated for OUD over an eight-year period in Ontario, Canada *[primary objective]*.
2. Explore sociodemographic and clinical characteristics associated with multimorbidity among people with OUD *[secondary objective]*.
3. Determine patterns of multimorbidity using HCA and K-means clustering techniques *[secondary objective]*.
4. Examine whether choice of clustering method affects the number of clusters and composition of multimorbidity patterns *[secondary objective]*.

## Materials and methods

### 1. Study design and setting

We used a retrospective single-cohort design for the current study, in which data from two observational studies were merged and linked to Ontario provincial health administrative databases. We adhered to the reporting standards outlined in the Reporting of studies Conducted using Observational Routinely-collected Data (RECORD) guidelines (*see AS1 File*) [31,32].

This study is part of our research group’s larger initiative to assess long-term outcomes of people with OUD, with all studies employing a similar single-cohort design to inform strategies for various aspects of care for this clinical population [33–37].

### 2. Study population

To identify people with OUD, we utilized data from two prospective cohort studies: the GENetics of Opioid Addiction (GENOA) study and the Pharmacogenetics of Opioid Substitution Treatment Response (POST) study. Both studies recruited people undergoing Opioid Agonist Treatment (OAT) for OUD at outpatient clinics across Ontario, Canada [38–40]. which are part of a centrally managed network run by the Canadian Addiction Treatment Centres. Inclusion and exclusion criteria have been previously described [38–42]. People with OUD were recruited into the GENOA study between 01/06/2011 and 30/04/2017, whereas the POST study recruited participants from 01/05/2018 to 30/04/2021. Led by the same research team, both studies followed identical protocols for participant recruitment and data collection, minimizing the potential for measurement error when combining cohort data. Ethical approval was granted by the Hamilton Integrated Research Ethics Board for both studies (GENOA project ID 11–056; POST project ID 4556; ICES linkage project IDs 12602 and 12767-C). During data collection, the research team collected identifying information, which was later used by the lead author to merge data from the GENOA and POST cohorts, excluding duplicate enrollments and retaining the most complete record for each individual (*n* = 272 excluded; S2 File, S1 Fig).

### 3. Data sources

The cohort data were linked with Ontario provincial administrative health data housed at ICES (formerly known as the Institute for Clinical Evaluative Sciences), an independent non-profit research institute operating under Ontario’s health information privacy law. ICES collects and analyzes health care and demographic data for health system evaluation and improvement without requiring consent. The individual-level cohort data were linked to unique encoded identifiers at ICES using deterministic linkage with Ontario health card numbers and dates of birth for participants enrolled in Ontario’s public health insurance program (>96% coverage). Exclusions were made for invalid linkage, lack of Ontario Health Insurance Program (OHIP) eligibility, or residence outside the province *(*see S2 File, S1 Fig). Data linkage included health records from ICES holdings five years prior to cohort enrollment and three years post-recruitment, with shorter follow-up for individuals censored due to death or relocation out of the province *(n* = 43*)*, or recruitment after April 1, 2021 (*n* = 40), as ICES data were available only up to March 31, 2022. Identifying information for participants was available to the ICES data linkage staff only during linkage and removed after successful linkage of participant-level data to administrative health records. ICES data was accessed from 01/12/2023 to 20/05/2024 to complete analyses. All database analyses were conducted in compliance with Ontario privacy legislation, authorized under section 45 of Ontario’s Personal Health Information Protection Act, without requiring review by a Research Ethics Board.

### 4. Baseline characteristics

Information on age and sex were sourced from the Registered Persons Database, a population-based registry managed by ICES. Additional demographic and clinical details from the GENOA and POST studies, collected at study entry, included: marital status (single or in a relationship), employment status (unemployed or employed), and type of OAT (methadone or buprenorphine). Patients on other treatments, i.e., heroin-assisted therapy, were included in the methadone group because both treatments involve full opioid agonists [43,44], whereas buprenorphine is a partial agonist [45]. Additionally, since the sample size for patients on heroin-assisted therapy was small *(n suppressed due to ICES privacy regulations)*, combining them with the larger methadone group likely had minimal impact on the overall analysis.

### 5. Definition of outcome

#### (i) Coding and selection of diseases.

We examined 16 chronic conditions, which were selected based on their high prevalence and economic burden [46–48], and have also been used in prior research assessing multimorbidity in ICES databases in Ontario [49–51]. A recent systematic review classified this definition of multimorbidity as being methodologically robust, based on the number and definitions of included conditions, use in prior literature, clear identification of International Classification of Diseases (ICD) diagnostic codes, and criterion and predictive validity [52]. Each condition was defined using health administrative data based on the International Classification of Diseases, Ninth (ICD-9) and Tenth Revision (ICD-10) criteria in CIHI-DAD or OHIP databases. When possible, we used validated ICES-derived cohorts or disease registries to ascertain presence of conditions, including rheumatoid arthritis, asthma, cancer, CHF, COPD, dementia, diabetes, hypertension, and renal disease. These cohorts have been typically defined based on a diagnostic code present on any hospital discharge (CIHI-DAD) or on two physician visits (OHIP) within a 2-period. We followed this definition to identify: acute myocardial infarction (AMI), osteo-arthritis and other arthritis *(presence of either or both these conditions constituted a count of 1 chronic condition)*, cardiac arrhythmia, coronary syndrome (excluding AMI, also known as ischemic heart disease), mood and anxiety disorders, osteoporosis, and stroke (excluding transient ischemic attack). Diagnostic codes may be found in S2 File, S1Table and have been used by prior Ontario-based observational studies assessing multimorbidity [49–51]. We slightly modified the definition of mental health conditions to include both mood and anxiety disorders, since these psychiatric conditions often co-occur both in the general population [53,54] and among people with OUD [55]. This comprehensive definition of mental health disorders has been used by a recent population-based study assessing multimorbidity in Ontario [56].

Each chronic condition was coded as a binary variable, indicating the absence or presence of the disease. We identified conditions present at the index date (recruitment into the cohort study) using a lookback window of 2 years, as well as the presence of conditions that developed over the 8-year study follow-up period.

#### (ii) Multimorbidity definition.

Multimorbidity was defined by a binary variable: the presence of 2 + chronic conditions from our list above. Although OUD is a chronic condition, it was excluded from the multimorbidity count because it was the exposure of interest and is not typically included in standard definitions of multimorbidity [49–51]. A similar approach to assess the outcome of multimorbidity among people with existing mental health conditions has been used by prior research [57]. Our primary outcome was the prevalence of multimorbidity over the entire 8-year period: 5 years pre-study entry and 3 years post-enrollment.

### 6. Statistical analysis

All analyses were conducted using Stata MP Version 15.1 [58], with Microsoft Excel used to create selected tables and figures. Descriptive statistics on baseline characteristics of patients, and on the prevalence of individual chronic conditions over the study period were summarized using frequencies and proportions for categorical data and means and standard deviations (s.d.) for continuous data.

#### (i) Prevalence of multimorbidity.

We estimated the prevalence of multimorbidity over eight years with a 95% confidence interval (CI). Prevalence was selected as the primary measure because multimorbidity refers to chronic conditions that often develop and persist over time, making it more relevant to capture the overall burden of these conditions within the population rather than the rate of new cases. This approach is typically used in multimorbidity research [49–51]. Additionally, we assessed prevalence both pre- and post-study entry, and in subgroups formed by age (<25 years, 25–54 years, 55 years and older (55+)), sex (males, females), and OAT type (methadone, buprenorphine). These age thresholds, derived from prior studies assessing OUD populations [59,60], allow for meaningful comparisons across developmental stages and life transitions, which are associated with varying risks of multimorbidity. Stratifying by sex and OAT type provides further insight into potential variations in risk for co-occurring chronic conditions within key demographic and clinical subgroups.

#### (ii) Factors associated with multimorbidity.

We then used modified Poisson regression models with robust variance estimators, with overall multimorbidity as the dependent variable, and explored its association with baseline sociodemographic and clinical characteristics including sex, age, marital status, employment status, and type of OAT over the eight-year study period. Factors were selected based on established relevance in prior research specific to populations with OUD [13,14] or as determined by clinicians on our team (ZS, TR, BBD). While dose and duration of OAT were not examined due to limited prior evidence on their relationship to multimorbidity, type of OAT was included. Methadone and buprenorphine are both recommended as first-line treatments for OUD in many clinical guidelines [61], but differ in their pharmacological profiles—methadone being associated with increased cardiac risks, for instance, which may result in differential patterns of multimorbidity [62,63]. We reported prevalence ratios (PRs) with 95% CIs for each factor.

#### (iii) Patterns of multimorbidity.

We identified disease patterns in the subset of participants who had multimorbidity (2 + chronic diseases) in our study sample using two approaches: (i) HCA and (ii) K-means clustering. Given our assessment of a particular segment of the general population, we opted to cluster diseases not patients in our study. Our approach has been deemed useful for revealing patterns of co-occurrence, with the ability to inform management of chronic disease for clinical decision-making contexts [64]. Both HCA and K-means clustering were employed to capture different dimensions of multimorbidity patterns. HCA was used to explore hierarchical relationships between diseases, providing an intuitive structure that reflects clinical co-occurrence patterns. This method allowed for the visualization of how conditions may cluster together, aligning with disease progression or co-occurrence in clinical practice. Additionally, K-means clustering was utilized to efficiently handle large datasets and reduce sensitivity to outliers, an advantage for population-based studies of multimorbidity patterns [20,21]. By incorporating both methods, we aimed to gain a comprehensive understanding of disease patterns, leveraging the strengths of each approach.

**(A) Hierarchical cluster analysis**: Hierarchical cluster analysis groups diagnoses into clusters based on their similarity, as measured by the Jaccard coefficient. This coefficient quantifies the similarity between two diagnoses by considering only the diagnoses present in at least one of any two patients. Since the optimal number of clusters was unknown, we employed agglomerative hierarchical methods, including: average linkage, Ward, complete linkage, single linkage, median linkage, weighted average linkage and centroid linkage. All, except Ward’s and average linkage, omitted two or more of the 16 diseases from the clustering solution. Thus, we selected the Ward method as the primary approach, since it minimizes within-cluster variance, and can identify meaningful clusters without biasing the results towards a particular cluster size, as with average linkage clustering. We conducted visual inspection of the dendrogram, and additionally examined the Caliński-Harabasz pseudo-F statistic, Duda–Hart Je(2)/Je(1) stopping-rule value and pseudo-T2 statistic. The criteria for selecting the number of clusters were: large Caliński–Harabasz pseudo-F values, large Duda–Hart Je(2)/Je(1) values, and small Duda–Hart pseudo-T2 values, which characterize distinct clustering [21,65]. If there were ties between number of clusters, we opted for the solution with a higher number of clusters. Additionally, we conducted a sensitivity analysis using average linkage, to assess the robustness of our findings.

We used Multidimensional Scaling (MDS) with two dimensions to uncover the inherent structure of distances between diseases identified in the cluster analysis. MDS maps observations to positions in a conceptual space, aiming to align distances between points in this space with the dissimilarities calculated in the cluster analysis. Specifically, classical MDS was conducted using the distance matrix derived from the cluster analysis, where the Jaccard coefficient served as the dissimilarity measure [66–68]. We visually depicted our findings from MDS using a conceptual map.

**(B) K-means analysis**: Prior to conducting K-means clustering, we applied multiple correspondence analysis (MCA) to our sample of patients with multimorbidity. MCA is often used to reveal the latent structures within binary or categorical data, i.e., diagnoses, facilitating the visualization of relationships among variables that might not be readily apparent from contingency tables. This method was used to transform binary diagnoses into continuous dimensions, enabling the direct representation of diseases in a geometric space [69]. The optimal number of dimensions were determined using a scree plot and dimensional plot. If there were discrepancies between these two plots, we opted for the solution with the maximum number of dimensions.

For the K-means clustering process, diseases were allocated into a pre-defined clusters based on the number of dimensions derived from MCA. The K-means algorithm involves several steps: (i) initially placing K points (centroids) into the geometric space to represent the initial groupings, (ii) assigning each disease to the nearest centroid, and (iii) recalculating the centroids’ positions iteratively until convergence is achieved, thereby resulting in distinct and homogeneous within-disease clusters and maximum heterogeneity between groups (diseases) [21]. We assessed the Caliński-Harabasz index value for the number of clusters in our grouping solution [21,65], and additionally depicted the inter- and intra-clustering patterns using a scatterplot matrix.

#### (iv) Comparison of multimorbidity patterns.

Both statistical expertise (MR, SP, LT) and clinical judgment (ZS, TR, BBD) were used to assess the utility of the final clustering solutions for each technique, based on patterns observed in prior research and clinical practice, in addition to consensus opinions of the study team. We summarized multimorbidity patterns obtained from each technique visually through tables and descriptively compared the number and composition of clusters across both methods.

## Results

### Study sample

The merged POST and GENOA study sample consisted of 3,486 participants. After exclusion of 56 participants, who were unable to be linked to ICES data holdings, our final study sample consisted of 3,430 people with OUD (see S2 File, S1 Fig).

The overall age of our sample was 40.0 years (s.d. = 11), with 55.9% of the participants being male (*n* = 1,916/3,430) while 44.14% of the sample were female (*n* = 1,514). The sociodemographic characteristics of the cohort separated by presence of overall multimorbidity are presented in Table 1. The majority of participants in our study were single (*n* = 2,399, 69.9%) and unemployed (*n* = 2,269, 66.2%). Most people with OUD were on methadone maintenance treatment (*n* = 1,879, 79.4%).

**Table 1:** Demographic and clinical characteristics (*n* = 3,430).

Demographic or clinical factor	People without multimorbidity (*n* = 2,316, *n*, %)	People with multimorbidity^1^ (*n* = 1,114, *n*, %)
**Sex**		
Male	1,339 (57.8)	577 (51.8)
Female	977 (42.2)	537 (48.2)
**Age** *(years)*		
<25	203 (8.8)	38 (3.4)
25-54	1,975 (85.3)	849 (76.2)
55+	138 (6.0)	227 (20.4)
**Age** *(years; mean, sd)*	36.74 (9.9)	43.70 (11.6)
**Marital status**		
Single	1,613 (69.6)	786 (70.6)
Married or common-law	703 (30.4)	328 (29.4)
**Employment status**		
Unemployed	1,435 (62.0)	834 (74.9)
Employed	878 (38.0)	280 (25.1)
**Type of OAT** ^2^		
Methadone	1,305 (79.6)	574 (79.0)
Buprenorphine	335 (20.4)	153 (21.0)

Abbreviations: OAT: opioid agonist therapy; sd: standard deviation.

Notes:

^1^overall multimorbidity during 8-year study period;

^2^*n* reduced due to missing responses.

### Prevalence of multimorbidity

Overall, 1,114 people with OUD (32.5%, 95% CI = 30.9, 34.1) had 2 + other co-occurring chronic conditions over the eight-year study period (see S2 File, S2 Table). The prevalence of multimorbidity was 22.2% (*n* = 761, 95% CI = 20.8, 23.6) in the five years before study recruitment, and 13.0% (*n* = 447, 95% CI = 11.9, 14.2) post study entry. The number and prevalence of individual chronic conditions for the entire study sample and those with multimorbidity have been summarized in S2 File, S3, S4 Tables and S2, S3 Figs.

### Factors associated with multimorbidity

Prevalence ratios summarizing the effect of sociodemographic and clinical factors on the prevalence of overall multimorbidity are presented in Table 2. People aged 55 + had over a three times greater prevalence of multimorbidity compared to those below 25 years of age (PR = 3.39, 95% CI = 2.36, 4.87). We did not find an association between marital status and multimorbidity (PR = 1.00, 95% CI = 0.88, 1.14). The estimate of the association between sex and multimorbidity was uncertain, indicating a range from a slight protective effect to an increased association, for males compared to females (PR = 1.07, 95% CI = 0.95, 1.64). Similarly, there was uncertainty in the association between OAT and prevalence of 2 + co-occurring chronic diseases, ranging from a small protective effect to an increased association, for people on buprenorphine, compared to those on methadone (PR = 1.09, 95% CI = 0.94, 1.26). People with OUD who were unemployed were also more likely to experience multimorbidity than those who were employed (PR = 1.31 95% CI = 1.13, 1,54).

**Table 2:** Prevalence ratios of demographic and clinical factors and their association with multimorbidity among people with opioid use disorder over the eight-year study period (*n* = 2,367).

Demographic or clinical factor	Prevalence Ratio (95% CI)^1^
**Age** *(years)*	
<25	*Reference*
25-54	1.66 (1.17, 2.37)
55+	3.39 (2.36, 4.87)
**Sex**	
Male	*Reference*
Female	1.07 (0.95, 1.20)
**Marital status**	
Married or common-law	*Reference*
Single	1.00 (0.88, 1.14)
**Employment status**	
Employed	*Reference*
Unemployed	1.31 (1.13, 1.54)
**Type of OAT** ^2^	
Methadone	*Reference*
Buprenorphine	1.09 (0.94, 1.26)

Abbreviations: CI: confidence interval; OAT: opioid agonist therapy.

Notes:

^1^overall multimorbidity during 8-year study period;

^2^*n* reduced due to missing responses.

### Patterns of multimorbidity

#### Hierarchical cluster analysis.

**Fig 1:**
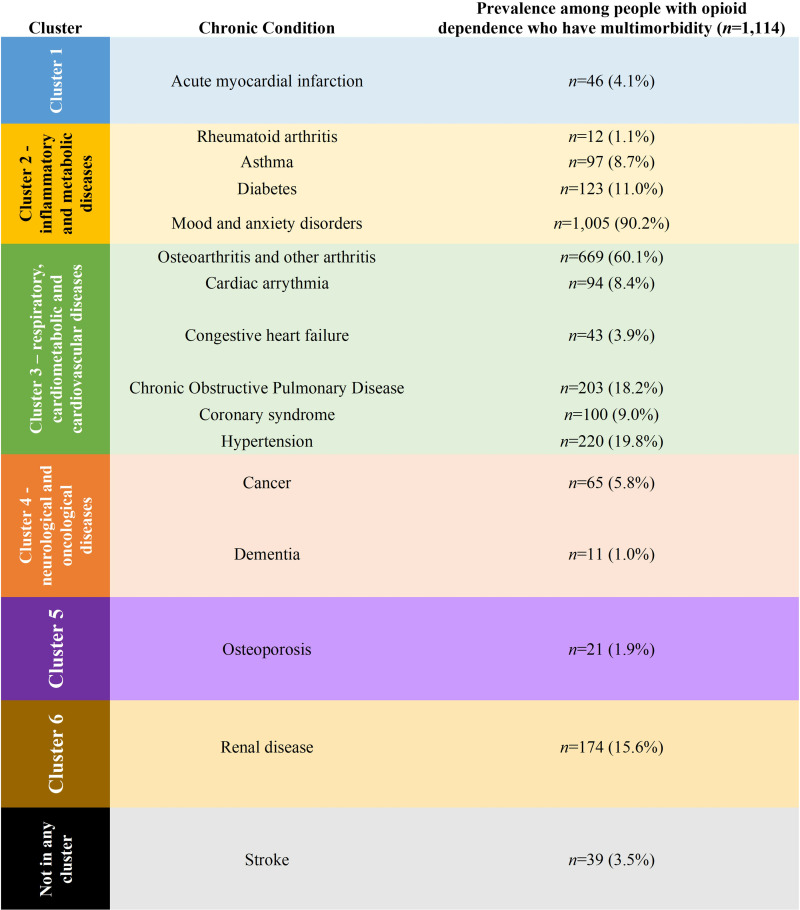
Clusters obtained from hierarchical cluster analysis with Ward’s linkage. Hierarchical cluster analysis with Ward’s linkage identified six distinct disease clusters, omitting stroke from the clustering algorithm.

**Fig 2:**
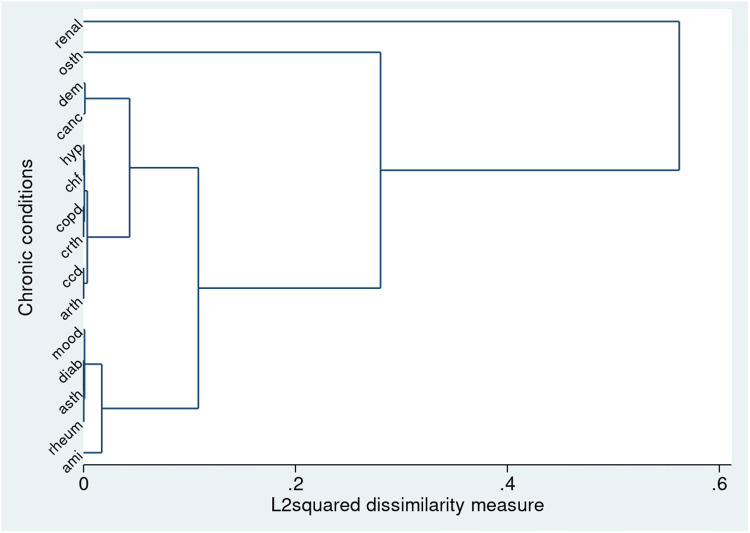
Dendrogram from hierarchical cluster analysis with Ward’s linkage. Abbreviations: ami: acute myocardial infarction; arth: osteoarthritis and other arthritis; asth: asthma; canc: cancer; ccd: coronary syndrome (ischemic heart disease); chf: congestive heart failure; copd: chronic obstructive pulmonary disease; crth: cardiac arrhythmia; dem: dementia; diab: diabetes; hyp: hypertension; mood: mood and anxiety disorders; osth: osteoporosis; renal: renal disease; rheum: rheumatoid arthritis; strk: stroke.

We identified 6 clusters grouping 15 diseases when using HCA with Ward’s linkage (see Fig 1 and S2 File, S5 Table). Clusters 1, 5 and 6 segregated AMI, osteoporosis, and renal disease into stand-alone clusters, suggesting distinct patterns of morbidity related to these diseases. Cluster 2 encompassed conditions with inflammatory and metabolic components, including rheumatoid arthritis, asthma, diabetes, and mood and anxiety disorders. Cluster 3 comprised of a variety of cardiometabolic and cardiovascular diseases such as osteoarthritis and other arthritis, cardiac arrhythmia, CHF, COPD, coronary syndrome, and hypertension. Cluster 4 grouped cancer and dementia, suggesting a clustering of chronic conditions associated with aging and malignancy. Notably, the presence of stroke did not align with any specific clusters, suggesting lack of alignment with the other chronic conditions in our dataset. A visual representation of these clusters is presented in a dendrogram in Fig 2 and an MDS scaling plot in S2 File, S4 Fig. We obtained similar findings from our sensitivity analysis using HCA with average linkage. This method grouped diseases into 5 clusters, comprising similar conditions as our primary analysis (see S2 File, S5 Fig and S5, S6 Tables).

#### K-means analysis.

**Fig 3:**
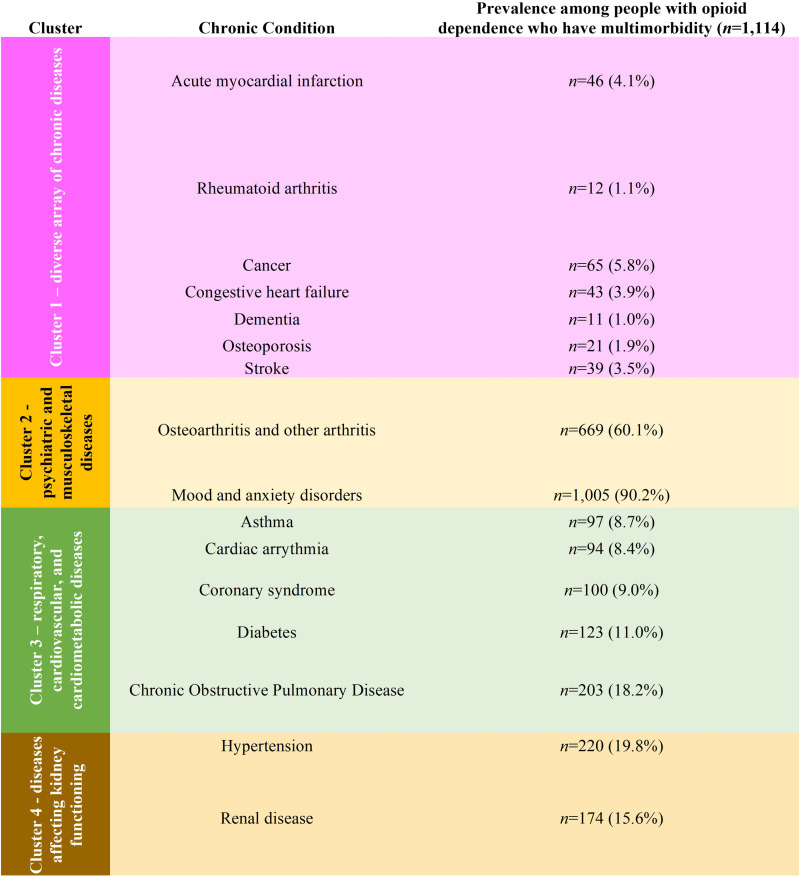
Clusters obtained from K-means clustering. K-means clustering identified four disease clusters and included stroke in the disease groupings.

Use of MCA identified as few as two (see S2 File, S6 Fig) and as many as four (see S2 File, S7 Fig) distinct dimensions. K-means clustering using the Caliński–Harabasz criterion to obtain four clusters was considered the optimal solution (see S2 File, S8 Fig), which have been presented in Fig 3. Cluster 1 grouped a diverse array of cardiovascular, musculoskeletal, neurological, and oncological disorders, whereas Cluster 2 was comprised of musculoskeletal and mental health ailments. Respiratory, cardiovascular, and cardiometabolic conditions were grouped together in Cluster 3. Finally, Cluster 4 grouped diseases affecting kidney functioning, i.e., hypertension and renal disease.

### Comparison of multimorbidity patterns

The HCA multimorbidity patterns could be interpreted more easily than the groupings obtained from K-means analysis, since it made more sense from a clinical standpoint, by the clustering of similar chronic diseases within each cluster. Both methods differed in the number of clusters, with HCA grouping the conditions into 6 clusters, and K-means forming 4 groups of chronic illnesses. Furthermore, the specific composition and organization of clusters differed between the two methods. For instance, AMI, renal disease, and osteoporosis were grouped with other conditions in K-means clustering, whereas each disease was assigned to a separate cluster in HCA. Nonetheless, there was some overlap in the clustering of diseases with similar clinical characteristics or underlying pathophysiological mechanisms, as demonstrated by the identification of cardiovascular, cardiometabolic, and respiratory diseases within Cluster 3 for both methods.

## Discussion

Our study highlights the substantial burden of multimorbidity experienced by people with OUD, with nearly one-third of the study population experiencing 2 + chronic diseases. We also found a notable age gradient, with adults aged 55 + exhibiting a higher prevalence of multimorbidity compared to those below 25 years of age. Furthermore, we identified both male sex and employment status as potential protective factors in the development of multiple co-occurring chronic conditions. While our cluster analytic techniques revealed distinct clusters of chronic illnesses, both methods identified groupings of cardiovascular, cardiometabolic, and respiratory diseases, reflecting the complex interplay of these diseases in people with OUD.

Our findings align with studies conducted in other jurisdictions, which have reported a significant increase in the incidence of multimorbidity among people with OUD compared to the general population [13], and identified patterns of cardiovascular and cardiometabolic diseases [14]. Furthermore, our findings suggest that both biological (i.e., age, sex) and socioeconomic (e.g., employment status) factors may play a role in shaping the health profiles of people with OUD, which has also been demonstrated by prior research [27].

There are four potential reasons for our findings. First, even among younger cohorts like those in our study sample, the burden of multiple chronic diseases remains substantial, indicating that OUD itself may contribute to the development of certain cardiometabolic complications that serve as risk factors for the chronic diseases included in our definition of multimorbidity. For instance, methadone treatment has been demonstrated to be associated with an increased risk of respiratory and cardiometabolic side effects, and increased rates of diabetes and gastrointestinal and liver cancer [70–73]. While the interplay between OUD, co-occurring chronic diseases, and withdrawal medications such as methadone is complex, it falls outside the primary scope of this study. Further research is needed to disentangle these relationships and assess their implications for clinical care and chronic disease patterns among people with OUD. Second, greater duration of OUD is associated with an increased risk for a comorbid physical or psychiatric illness [74], with aging increasing the risk for developing chronic diseases [75–78]. Third, psychiatric conditions such as mood and anxiety disorders are more pervasive among females as compared to males with OUD in treatment and non-treatment samples [14,79,80], thereby resulting in a higher prevalence of multimorbidity among females in our study. Fourth, the association between employment status and multimorbidity may reflect broader socioeconomic factors, as people with OUD who experience a higher burden of chronic diseases are more likely to rely on social assistance programs and consequently report being unemployed, including those on disability due to health conditions [81].

It is challenging to compare the findings from our comparative cluster analyses to other studies, due to variations in methods employed, data sources, and grouping of diseases vs. patients. In particular, we examined only a segment of the general population who are high health-care users [13,82], whereas most studies have assessed the general populations residing in their respective jurisdictions [20] or solely employed one approach for determining multimorbidity patterns [25,27]. Nonetheless, the discrepancies between the obtained disease clusters from HCA and K-means analysis echoes findings from a prior study comparing HCA with EFA, which demonstrated that no grouping solution contained exactly the same set of diseases [20].

The disparities in clustering solutions may be attributed to fundamental differences in the algorithmic approaches and handling of data characteristics between the two methods. As a hierarchical method, HCA with Ward’s linkage merges clusters iteratively to minimize within-cluster variance and considers the entire dataset during clustering, allowing for effective handling of both continuous and binary data [21]. Conversely, K-means clustering partitions the dataset into a predefined number of clusters based on Euclidean distance, which may lead to sensitivity to initial cluster centroids and local optima [21]. Additionally, the transformation of binary data into continuous variables through MCA (a data reduction technique) prior to employing K-means clustering may have introduced complexity and affect the resulting interpretation of distances and centroids.

While we observe discrepancies between HCA and K-means clustering solutions, these differences emphasize the need to consider the implications of each method in clinical practice. Despite the methodological variations, both approaches identified patterns of multimorbidity that carry significant implications for healthcare delivery. However, HCA’s dendrogram structure provided a more transparent view of how certain conditions, such as AMI, osteoporosis, and renal disease, aligned (or did not align) with larger clusters. This step-by-step merging process highlights potential shared aetiologies and clinical overlap, thereby enhancing interpretability for clinicians and researchers. Given these observations, in addition to the more intuitive interpretation of cluster structure obtained from the dendrogram in HCA with Ward’s linkage, we will prioritize the disease groupings obtained from this method developing actionable insights for knowledge users.

### Implications for decision-making contexts

These multimorbidity patterns have direct implications for clinical care, particularly in the treatment of people with OUD. First, three clusters consisted of stand-alone diseases – AMI, osteoporosis, and renal disease, whereas stroke was left out of the clustering solution altogether, suggesting lack of etiological and pathophysiological overlap with the other conditions included in the clusters, and distinct patterns of morbidity related to these chronic diseases among people with OUD. Regardless, clinicians caring for people with OUD, particularly those who are 55 + , should screen for presence of these individual conditions, and adjust treatment options accordingly [59,83]. For instance, patients with AMI may be prescribed angiotensin-converting enzyme inhibitors (ACEIs) to manage cardiovascular risk factors. Clinicians should monitor for interactions with opioids, and further exacerbation of hypotension or respiratory depression, which are already side-effects of treatment with clonidine and methadone [84–86].

Second, the frequent overlap of cardiovascular, cardiometabolic, and respiratory diseases identified by both methods highlight the necessity of tailoring care for this clinical population. These clusters may share common physiological pathways, such as chronic inflammation, metabolic dysregulation, and compromised respiratory function [87,88]—factors potentially exacerbated by chronic opioid use [89].Thus, people with cardiac arrhythmias, CHF, and coronary disease may require antiarrhythmic agents, diuretics or ACEIs. Close monitoring for electrolyte abnormalities and renal function is essential when using these medications concurrently with opioids, as dehydration and renal impairment can potentiate opioid-related adverse effects [90–92]. Furthermore, people with arthritis and hypertension may use non-steroidal anti-inflammatory drugs (NSAIDs) and antihypertensive medications. Clinicians should monitor for interactions with opioids, as NSAIDs can increase the risk of gastrointestinal bleeding and impair renal function [93,94], while certain antihypertensive agents may potentiate the hypotensive effects of opioids [95].

While the multimorbidity patterns obtained by HCA and K-means analysis did not perfectly align, identification of shared disease pairs by both approaches highlights that presence of even one chronic illness is often linked to other conditions among people with OUD. For example, people in Cluster 2 presenting with rheumatoid arthritis, asthma, diabetes, and mood and anxiety disorders may necessitate a collaborative approach involving psychiatrists, endocrinologists, rheumatologists, and primary care physicians. This interdisciplinary team can optimize cardiovascular health while addressing symptoms associated with OUD. Effective care coordination among healthcare providers from various specialties is paramount to ensure integrated and comprehensive management for patients with OUD who have other chronic comorbidities [78,96]. Clinicians can also empower patients by offering education on the interplay between OUD and comorbidities, promoting lifestyle modifications, encouraging medication adherence, and imparting self-management strategies. Additionally, peer support groups and community resources may serve as invaluable assets in fostering resilience and facilitating recovery among people with OUD facing multiple health challenges [97–99].

The practical applications of these findings are strengthened by the robust methodological approach used in this study. However, it is important to consider several limitations when interpreting these results and their potential for guiding clinical decision-making.

### Strengths and limitations

We conducted the first study, to our knowledge, on multimorbidity among people with OUD in a Canadian setting and utilized a large cohort of people receiving treatment for this disorder over an eight-year period. Linkage of data from observational studies provincial health administrative databases enabled us to capture a broad spectrum of chronic conditions among our study sample. Furthermore, the use of both hierarchical cluster analysis and K-means clustering techniques provided a nuanced exploration of multimorbidity patterns, enhancing the robustness and reliability of our findings.

However, our study is not without limitations. First, our reliance on existing health administrative data limits our analyses to the conditions which are able to be identified using physician billings and/or hospitalisation records. Specifically, we lacked information on liver disease, which has demonstrated to be highly prevalent among people with OUD receiving methadone treatment [100], suggesting that we may have underestimated the prevalence of multimorbidity among our study sample. Second, our study follow-up was eight years, which is novel for research on chronic diseases in this clinical population [13,14]. However, a recent validation study assessing different follow-up periods, suggests that a minimum lookback window of ten years is required for stable estimation of multimorbidity prevalence, which should be considered when interpreting our findings [101]. Third our lack of comparator group precludes our ability to infer incidence, namely that of multimorbidity among people with OUD relative to those without OUD, which is critical for contextualizing the burden of multimorbidity. Fourth, part of our study period (2020–2021) overlapped with the COVID-19 pandemic; however, because our focus was on longer-term prevalence (rather than incidence) of multimorbid conditions, we could not quantify the pandemic’s effect on healthcare access or OUD treatment retention. Future research incorporating pandemic-specific disruptions is warranted. Fifth, our small sample size limits our ability to detect sex-specific clustering of chronic diseases, which may be important for delivering tailored care to this clinical population. For instance, prior studies have found that females with OUD exhibit patterns of mood and anxiety disorders, whereas patterns of cancer, diabetes, and renal disease have been seen in males with OUD [14,79]. Sixth, our approach to measuring multimorbidity may have influenced our findings. We identified chronic illnesses based on simple counts in health administrative data and treated all diseases equally in our count of multiple chronic conditions. This limits our ability to differentiate conditions based on severity or associated mortality risk, for instance through tools like the Charlson Comorbidity Index [102]. However, previous research has demonstrated that simple counts using health administrative data can accurately capture chronic conditions [103], and have predictive validity [104,105], Thus, we do not believe this to be a significant limitation of our study. Seventh, our study sample was limited to individuals receiving OAT in outpatient clinics, thereby excluding individuals with OUD who may not be engaged in treatment or receiving care in other settings. Furthermore, our findings, drawn from a single Canadian province (Ontario), may not fully generalize to different healthcare systems or geographical contexts, where baseline demographics and treatment practices can differ [106]. Patients not currently engaged in treatment may be impacted by even more chronic diseases, or experience different conditions altogether [107,108]. Thus, findings from our study may not be generalizable to all populations with OUD. While these limitations provide important context, they also open up avenues for future research to address these gaps.

### Future directions for research

There are several considerations for researchers who aim to study multimorbidity among populations with OUD. First, to better understand the incidence of multimorbidity, studies should incorporate cohort designs with comparison groups to determine incidence and assess sex-specific patterns of multiple chronic conditions, as demonstrated by prior research [14,79]. Additionally, future studies should examine the role of medication duration and its potential influence on the development of chronic conditions among people with OUD. Second, the role of sociodemographic characteristics in the development of multimorbidity among people with and without OUD should be explored, considering factors such as age, sex, and employment status [27]. Third, external validation of cluster solutions (e.g., testing on different OUD cohorts or through prospective clinical assessments) would further enhance reliability and real-world applicability. Finally, the choice of clustering method significantly influences the identification and interpretation of multimorbidity patterns. Thus, researchers should employ multiple cluster analytic techniques and compare findings from each method to ensure robustness and enhance reliability of results. While we interpreted clusters obtained from HCA, it is important to acknowledge the limitations of this method, including the exclusion of certain diseases like stroke from clustering solutions and the presence of isolated disease clusters, which may conflict with the concept of multimorbidity involving multiple diseases per cluster. Given these potential limitations of HCA, future research should explore the combination of methods, such as EFA and K-means, to obtain a more nuanced understanding of multimorbidity.

## Conclusions

In conclusion, our study highlighted the substantial burden of multimorbidity among individuals with OUD in Ontario, Canada, with nearly one-third experiencing 2 + chronic conditions. We identified age, sex, and employment status as potential determinants of multimorbidity in this population, highlighting the complex interplay of biological and socioeconomic factors in shaping health outcomes. Our findings also revealed distinct patterns of multimorbidity, with hierarchical cluster analysis and K-means clustering techniques offering complementary insights into disease groupings. Moving forward, addressing multimorbidity among individuals with OUD requires tailored, interdisciplinary approaches that consider both the clinical complexity of co-occurring conditions and the broader social determinants of health.

**What is new?**

**Key findings**

• Our study found that nearly one-third (32.5%) of people with opioid use disorder (OUD) in Ontario experienced two or more (2+) chronic conditions (multimorbidity) over an eight-year period.

**What this adds to what is known?**

- This is the first Canadian study to quantify the high prevalence of multimorbidity in people with OUD.
- This study is methodologically novel by use of both hierarchical and K-means clustering techniques to analyze patterns of 2 + multiple chronic conditions.

**What are the implications and what should change now?**

- Identified clusters of cardiovascular, cardiometabolic, and respiratory diseases necessitates tailored care for people with OUD, with consideration of medication interaction and coordinated healthcare delivery.
- Future research should employ a combination of clustering techniques to provide a more comprehensive understanding of multimorbidity patterns.

## Supporting information

S1 File RECORD checklist. (DOCX)

S2 File Supplementary tables and figures. (DOCX)

S3 File Visual abstract (TIF)
