# Supplementary material for: Comparative analysis of methods for identifying multimorbidity patterns among people with opioid use disorder: A retrospective single-cohort study
Source: PLoS One. 2025 Jun 12;20(6):e0324548. doi: 10.1371/journal.pone.0324548 (PMC12162124; doi:10.1371/journal.pone.0324548)
Supplement: S2 File — (DOCX) [file pone.0324548.s002.docx]

S2 File: Supplementary tables and figures

Comparative analysis of methods for identifying multimorbidity patterns among people with opioid use disorder: A retrospective single-cohort study

Contents

[Figure S1: Study flow diagram 2](#_Toc187491558)

[Table S1: ICES diagnostic codes for chronic conditions included in definition of multimorbidity 3](#_Toc187491559)

[Table S2: Prevalence of multimorbidity among subgroups of people with opioid dependence (*n*=3,430) 5](#_Toc187491560)

[Table S3: Number of chronic conditions in people with opioid dependence over eight-year follow-up (*n*=3,430) 6](#_Toc187491561)

[Figure S2: Prevalence of individual chronic conditions in people with opioid dependence over eight-year follow-up (*n*=3,430) 7](#_Toc187491562)

[Table S4: Number of chronic conditions in people with opioid dependence with multimorbidity over eight-year follow-up (*n*=1,114) 8](#_Toc187491563)

[Figure S3: Prevalence of individual chronic conditions in people with opioid dependence with multimorbidity over eight-year follow-up (*n*=1,114) 9](#_Toc187491564)

[Table S5: Stopping rules for hierarchical cluster analysis 10](#_Toc187491565)

[Figure S4: Multidimensional scaling plot 11](#_Toc187491566)

[Figure S5: Dendrogram from hierarchical cluster analysis with average linkage 12](#_Toc187491567)

[Table S6: Clusters obtained from hierarchical cluster analysis with average linkage 13](#_Toc187491568)

[Figure S6: Scree plot from multiple correspondence analysis 14](#_Toc187491569)

[Figure S7: Dimensional plot from multiple correspondence analysis 15](#_Toc187491570)

[Figure S8: Scatterplot matrix from K-means clustering 16](#_Toc187491571)

# Figure S1: Study flow diagram

Final GENOA and POST Cohort, with duplicates excluded

*n* = 3,486

Exclusion due to invalid linkage to ICES holdings

*n* = 23

Exclusion due to death date before index

*n* = 8

Exclusion due to no OHIP eligibility or out of Province at index

*n* = 25

Study sample successfully linked with ICES holdings and in analysis

*n* = 3,430

Total GENOA and POST Cohorts

*n* = 3,758

Exclusion due to duplicate enrolment

*n* = 272

***Abbreviations:*** GENOA: Genetics of Opioid Addiction study; OHIP: Ontario Health Insurance Program; POST: Pharmacogenetics of Opioid Substitution Treatment Response study

# Table S1: ICES diagnostic codes for chronic conditions included in definition of multimorbidity

| **Conditions in multimorbidity definition** | **Diagnostic codes** | **Databases** |
| --- | --- | --- |
| Acute myocardial infarction (AMI)^1^ | ***1 hospital discharge (CIHI-DAD) or 2 physician visits (OHIP)***  ***within a 2-year period***  ICD-9/OHIP: 410  ICD-10: I21 | DAD, OHIP |
| Osteo- and other arthritis  (A) Osteoarthritis  (B) Other Arthritis (includes Synovitis, Fibrositis, Connective tissue disorders, Ankylosing spondylitis, Gout Traumatic arthritis, pyogenic arthritis, Joint derangement, Dupuytren’s contracture, Other MSK disorders) | ***1 hospital discharge (CIHI-DAD) or 2 physician visits (OHIP)***  ***within a 2-year period***  (A)  ICD-9: 715  ICD-10: M15-M19  (B)  ICD-9: 727, 729, 710, 720, 274, 716, 711, 718, 728, 739  ICD-10: M00-M03, M07, M10, M11-M14, M20-M25, M30-M36, M65-M69, M7X | DAD, OHIP |
| Rheumatoid arthritis | ICES-derived cohort | *ORAD: Ontario Rheumatoid Arthritis Database |
| Asthma | ICES-derived cohort | ASTHMA: Ontario Asthma dataset |
| (all) Cancers | ICES disease registry | *OCR: Ontario Cancer Registry |
| Cardiac Arrhythmia | ***1 hospital discharge (CIHI-DAD) or 2 physician visits (OHIP)***  ***within a 2-year period***  ICD-9/OHIP: 427 (OHIP) / 4273 (DAD)  ICD-10: I480, I481 | DAD, OHIP |
| Congestive Heart Failure | ICES-derived cohort | CHF: Congestive Heart Failure |
| Chronic Obstructive Pulmonary Disease | ICES-derived cohort | COPD: Chronic Obstructive Pulmonary Disease |
| Coronary syndrome (excluding AMI) *(ischemic heart disease)* | ***1 hospital discharge (CIHI-DAD) or 2 physician visits (OHIP)***  ***within a 2-year period***  ICD-9/OHIP: 411-414  ICD-10: I20, I22-I25 | DAD, OHIP |
| Dementia | ICES-derived cohort | DEMENTIA: Ontario Dementia Database |
| Diabetes | ICES-derived cohort | *ODD: Ontario Diabetes Dataset |
| Hypertension | ICES-derived cohort | HYPER: Ontario Hypertension dataset |
| Mood and anxiety disorders | ***1 hospital discharge (CIHI-DAD) or 2 physician visits (OHIP) within a 2-year period***  **ICD-9/OHIP:** 296, 300, 309, 311  **ICD-10:** F30-F33, F341, F348, F349, F38-F42, F431, F432, F438, F44, F45.0, F45.1, F45.2, F48, F530, F68.0, F930, F99 | DAD, OHIP, OMHRS |
| Osteoporosis | ***1 hospital discharge (CIHI-DAD) or 2 physician visits (OHIP)***  ***within a 2-year period***  ICD-9/OHIP: 733  ICD-10: M81, M82 | DAD, OHIP |
| Renal disease | ICES-derived cohort | CORR: Canadian Organ Replacement Registry |
| Stroke (excluding transient ischemic attack) | ***1 hospital discharge (CIHI-DAD) or 2 physician visits (OHIP)***  ***within a 2-year period***  ICD-9/OHIP: 430, 431, 432, 434, 436  ICD-10: I60-I64 | DAD, OHIP |

***Abbreviations:*** CIHI-DAD: Canadian Institute for Health Information Discharge Abstract Database; ICD: International Classification of Diseases (Ninth and Tenth Revisions); OHIP: Ontario Health Insurance Plan; OMHRS: The Ontario Mental Health Reporting System.

**References:** ^1^Austin PC, Daly PA, Tu JV. A multicenter study of the coding accuracy of hospital discharge administrative data for patients admitted to cardiac care units in Ontario. American Heart Journal 2002;144:290–6.

# Table S2: Prevalence of multimorbidity among subgroups of people with opioid dependence (*n*=3,430)

| **Subgroup** | **Prevalence (*n*, %, CI)** |
| --- | --- |
| **Time**  5-years prior to study entry  3-years post study entry  Overall (throughout 8-year study period) | 761 (22.2%, 95% CI: 20.8, 23.6%)  447 (13.0%, 95% CI: 11.9, 14.2%)  1,114 (32.5%, 95% CI: 30.9, 34.1%) |
| **Sex**  Male  Female | 577 (30.1%, 95% CI: 28.1, 32.2%)  537 (35.5%, 95% CI: 33.1, 37.9%) |
| **Age (years)**  <25  25-54  55+ | 38 (15.7%; 95% CI: 11.4, 21.0%)  849 (30.1%; 95% CI: 28.4, 31.8%)  227 (62.2%; 95% CI: 57.0, 67.2%) |
| **Type of OAT**^1^  Methadone  Buprenorphine | 574 (30.6%; 95% CI: 28.5, 32.7%)  153 (31.4%; 95% CI: 27.3, 35.7%) |

**Abbreviations:** CI: confidence interval; OAT: opioid agonist therapy
**Notes:** ^1^*n* reduced due to missing responses

# Table S3: Number of chronic conditions in people with opioid dependence over eight-year follow-up (*n*=3,430)

| **Number of Chronic Conditions** | **Prevalence (*n*, %)** |
| --- | --- |
| 0 | 768 (22.4) |
| 1 | 1548 (45.1) |
| 2 | 695 (20.3) |
| 3 | 249 (7.3) |
| 4 | 100 (2.9) |
| 5 | 45 (1.3) |
| 6+ | 25 (0.7) |

# Figure S2: Prevalence of individual chronic conditions in people with opioid dependence over eight-year follow-up (*n*=3,430)


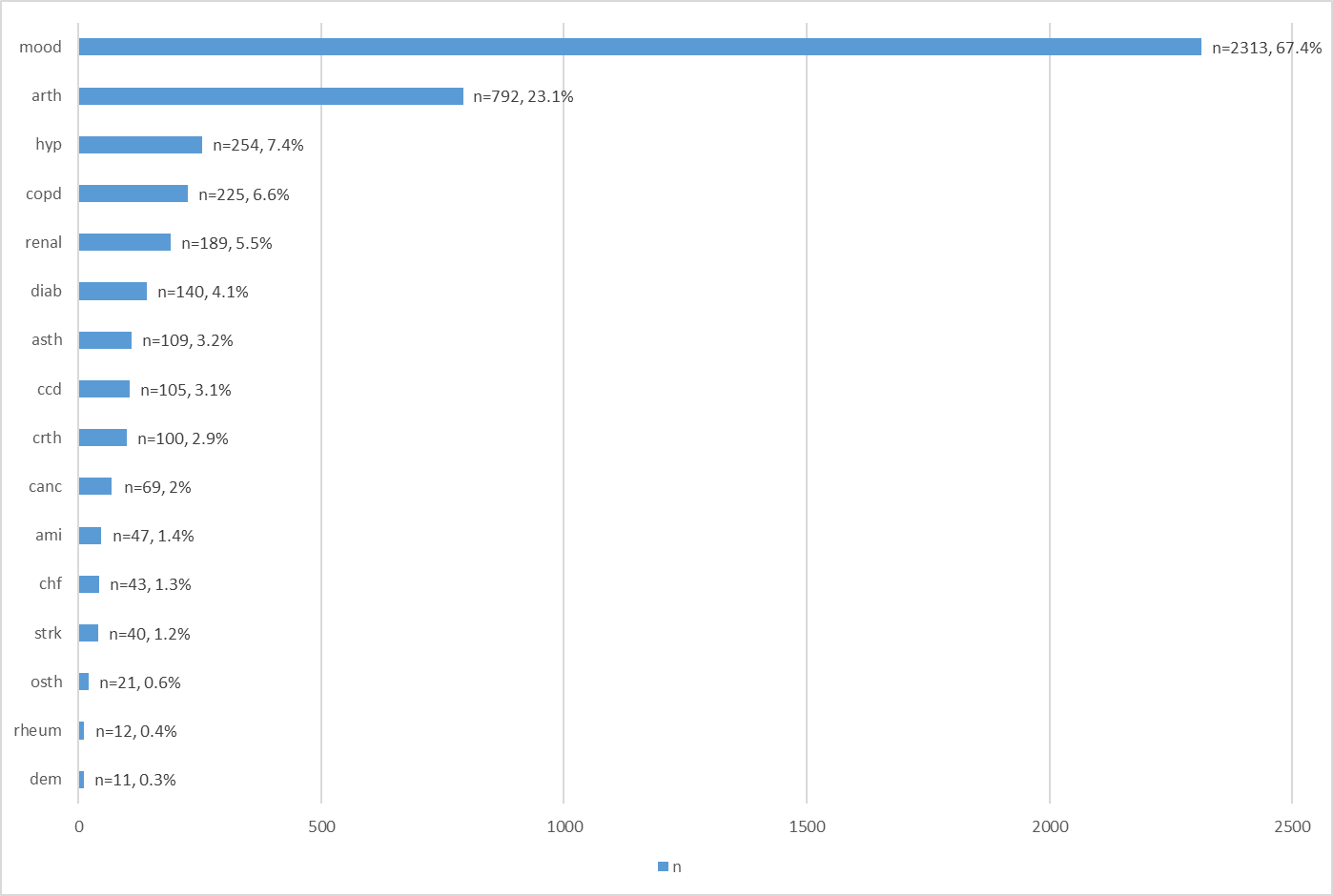


**Abbreviations:** ami: acute myocardial infarction; arth: osteoarthritis and other arthritis; asth: asthma; canc: cancer; ccd: coronary syndrome (ischemic heart disease); chf: congestive heart failure; copd: chronic obstructive pulmonary disease; crth: cardiac arrhythmia; dem: dementia; diab: diabetes; hyp: hypertension; mood: mood and anxiety disorders; osth: osteoporosis; renal: renal disease; rheum: rheumatoid arthritis; strk: stroke

# Table S4: Number of chronic conditions in people with opioid dependence with multimorbidity over eight-year follow-up (*n*=1,114)

| **Number of Chronic Conditions** | **Prevalence (*n*, %)** |
| --- | --- |
| 2 | 695 (62.4) |
| 3 | 249 (22.4) |
| 4 | 100 (9.0) |
| 5 | 45 (4.0) |
| 6+ | 25 (2.2) |

# Figure S3: Prevalence of individual chronic conditions in people with opioid dependence with multimorbidity over eight-year follow-up (*n*=1,114)


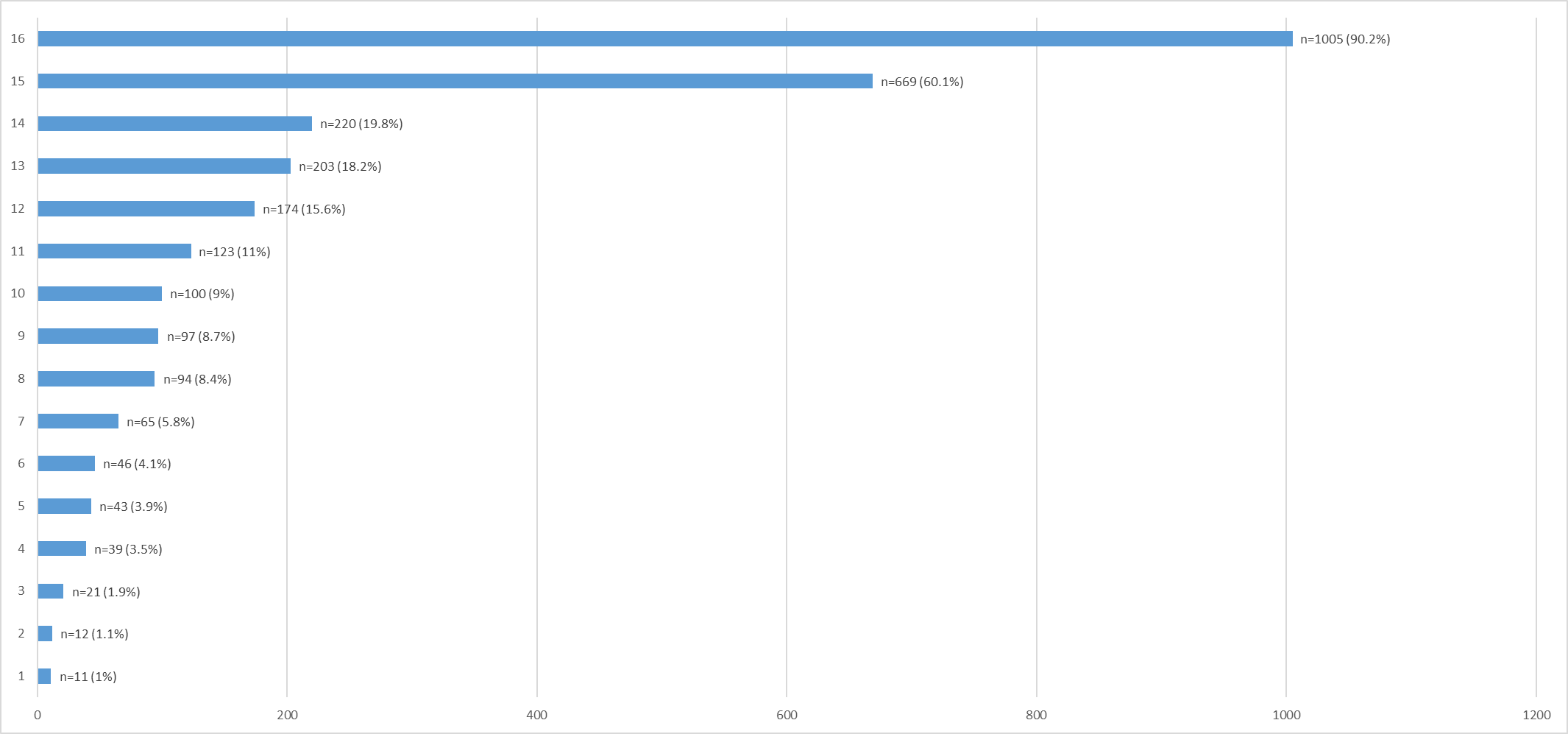


**Abbreviations:** ami: acute myocardial infarction; arth: osteoarthritis and other arthritis; asth: asthma; canc: cancer; ccd: coronary syndrome (ischemic heart disease); chf: congestive heart failure; copd: chronic obstructive pulmonary disease; crth: cardiac arrhythmia; dem: dementia; diab: diabetes; hyp: hypertension; mood: mood and anxiety disorders; osth: osteoporosis; renal: renal disease; rheum: rheumatoid arthritis; strk: stroke

# Table S5: Stopping rules for hierarchical cluster analysis

| **Ward’s linkage** | | | |
| --- | --- | --- | --- |
| **Number of clusters** | **Duda/Hart** | | **Caliński-Harabasz** **pseudo-F** |
|  | **Je (2)/ J2 (1)** | **pseudo T-squared** |  |
| 3 | 0.3835 | 17.68 | 28.77 |
| 4 | 0.1152 | 46.08 | 51.75 |
| 5 | 0.0586 | 48.19 | 103.82 |
| 6 | 0.207 | 15.32 | 270.68 |
| **Average linkage** | | | |
| **Number of clusters** | **Duda/Hart** | | **Caliński-Harabasz** **pseudo-F** |
|  | **Je (2)/ J2 (1)** | **pseudo T-squared** |  |
| 3 | 0.174 | 52.23 | 54.84 |
| 4 | 0.2514 | 11.91 | 210.04 |
| 5 | 0.2407 | 15.77 | 241.88 |
| 6 | 0.0278 | 34.93 | 423.87 |

# Figure S4: Multidimensional scaling plot


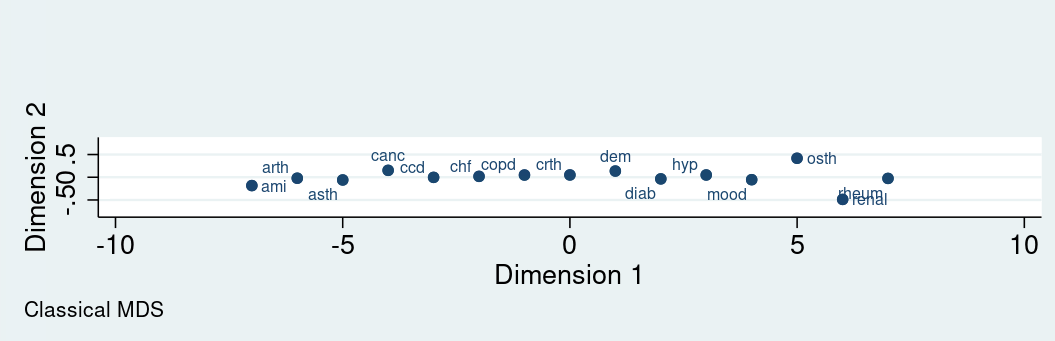


**Abbreviations:** ami: acute myocardial infarction; arth: osteoarthritis and other arthritis; asth: asthma; canc: cancer; ccd: coronary syndrome (ischemic heart disease); chf: congestive heart failure; copd: chronic obstructive pulmonary disease; crth: cardiac arrhythmia; dem: dementia; diab: diabetes; hyp: hypertension; mood: mood and anxiety disorders; osth: osteoporosis; renal: renal disease; rheum: rheumatoid arthritis; strk: stroke

# Figure S5: Dendrogram from hierarchical cluster analysis with average linkage


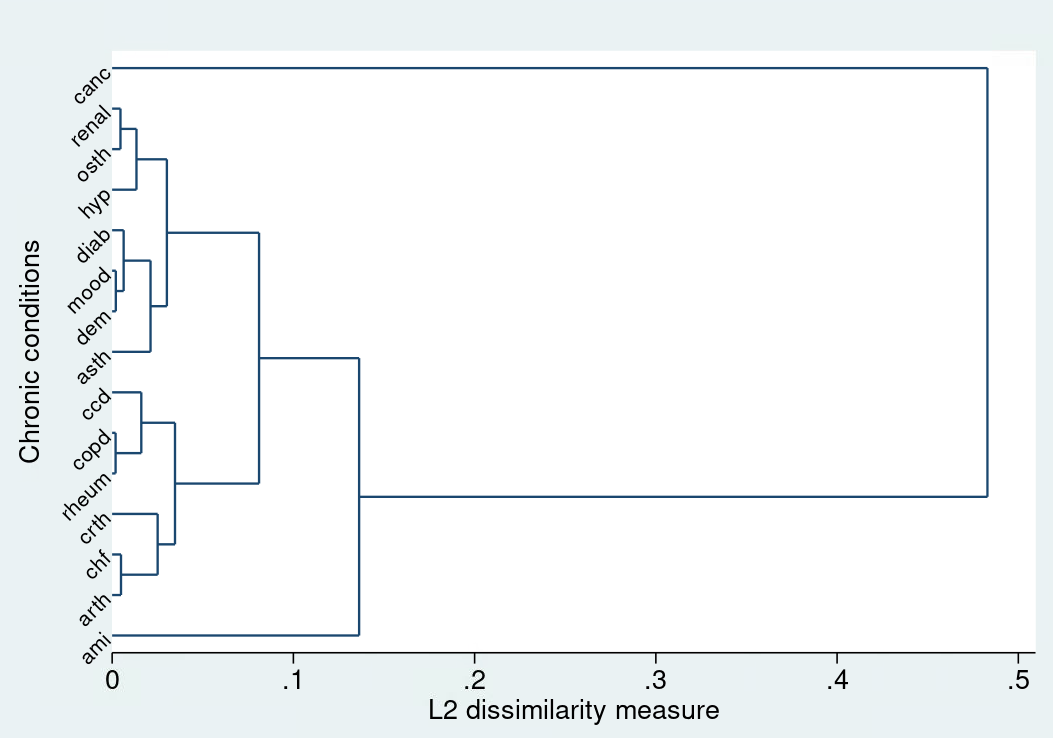


**Abbreviations:** ami: acute myocardial infarction; arth: osteoarthritis and other arthritis; asth: asthma; canc: cancer; ccd: coronary syndrome (ischemic heart disease); chf: congestive heart failure; copd: chronic obstructive pulmonary disease; crth: cardiac arrhythmia; dem: dementia; diab: diabetes; hyp: hypertension; mood: mood and anxiety disorders; osth: osteoporosis; renal: renal disease; rheum: rheumatoid arthritis; strk: stroke

# Table S6: Clusters obtained from hierarchical cluster analysis with average linkage

| **Cluster** | **Chronic Condition** | **Prevalence among people with opioid dependence who have multimorbidity (*n*=1,114)** |
| --- | --- | --- |
| **Cluster 1** | Acute myocardial infarction | *n*=46 (4.1%) |
| **Cluster 2 - musculoskeletal and cardiovascular diseases** | Osteoarthritis and other arthritis | *n*=669 (60.1%) |
|  | Cardiac arrythmia | *n*=94 (8.4%) |
|  | Congestive heart failure | *n*=43 (3.9%) |
| **Cluster 3 - autoimmune, respiratory, and cardiovascular diseases** | Rheumatoid arthritis | *n*=12 (1.1%) |
|  | Chronic Obstructive Pulmonary Disease | *n*=203 (18.2%) |
|  | Coronary syndrome | *n*=100 (9.0%) |
| **Cluster 4 - diverse group of diseases (metabolic, neurological, and psychiatric)** | Asthma | *n*=97 (8.7%) |
|  | Dementia | *n*=11 (1.0%) |
|  | Diabetes | *n*=123 (11.0%) |
|  | Hypertension | *n*=220 (19.8%) |
|  | Mood and anxiety disorders | *n*=1005 (90.2%) |
|  | Osteoporosis | *n*=21 (1.9%) |
|  | Renal disease | *n*=174 (15.6%) |
| **Cluster 5** | Cancer | *n*=65 (5.8%) |
| **Not in any cluster** | Stroke | *n*=39 (3.5%) |

# Figure S6: Scree plot from multiple correspondence analysis


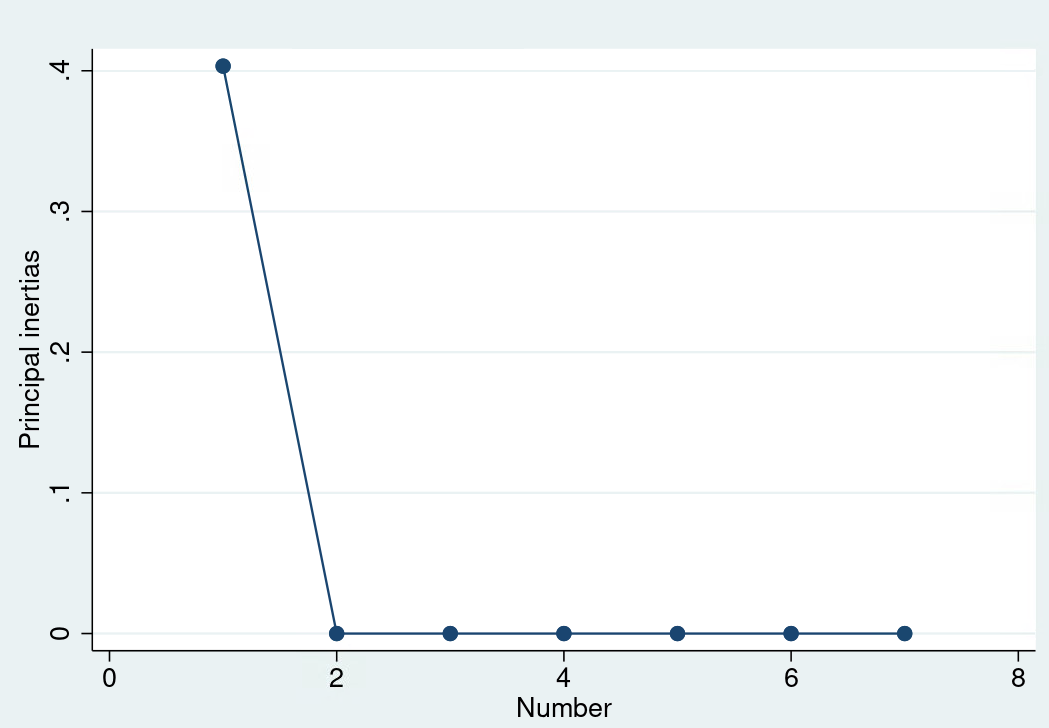


# Figure S7: Dimensional plot from multiple correspondence analysis


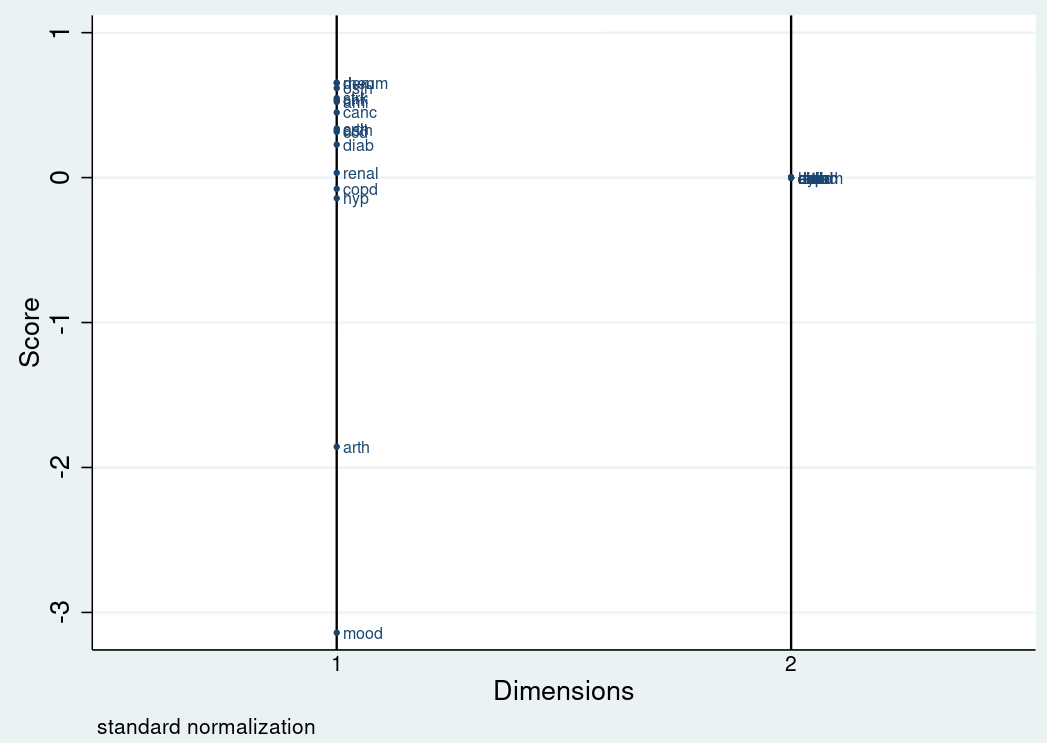


**Abbreviations:** ami: acute myocardial infarction; arth: osteoarthritis and other arthritis; asth: asthma; canc: cancer; ccd: coronary syndrome (ischemic heart disease); chf: congestive heart failure; copd: chronic obstructive pulmonary disease; crth: cardiac arrhythmia; dem: dementia; diab: diabetes; hyp: hypertension; mood: mood and anxiety disorders; osth: osteoporosis; renal: renal disease; rheum: rheumatoid arthritis; strk: stroke

# Figure S8: Scatterplot matrix from K-means clustering


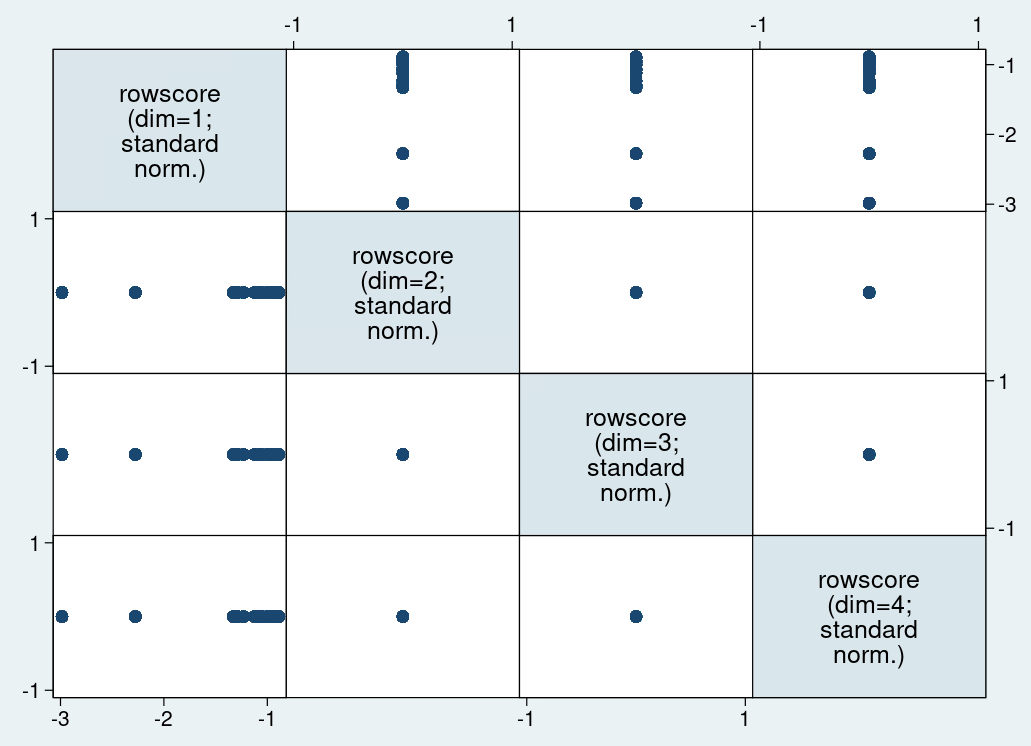


**Notes:** Stopping rule value: Caliński-Harabasz pseudo-F = 8252.75
