# Supplementary figures and images for: Comparative analysis of methods for identifying multimorbidity patterns among people with opioid use disorder: A retrospective single-cohort study
Source: PLoS One. 2025 Jun 12;20(6):e0324548. doi: 10.1371/journal.pone.0324548 (PMC12162124; doi:10.1371/journal.pone.0324548)

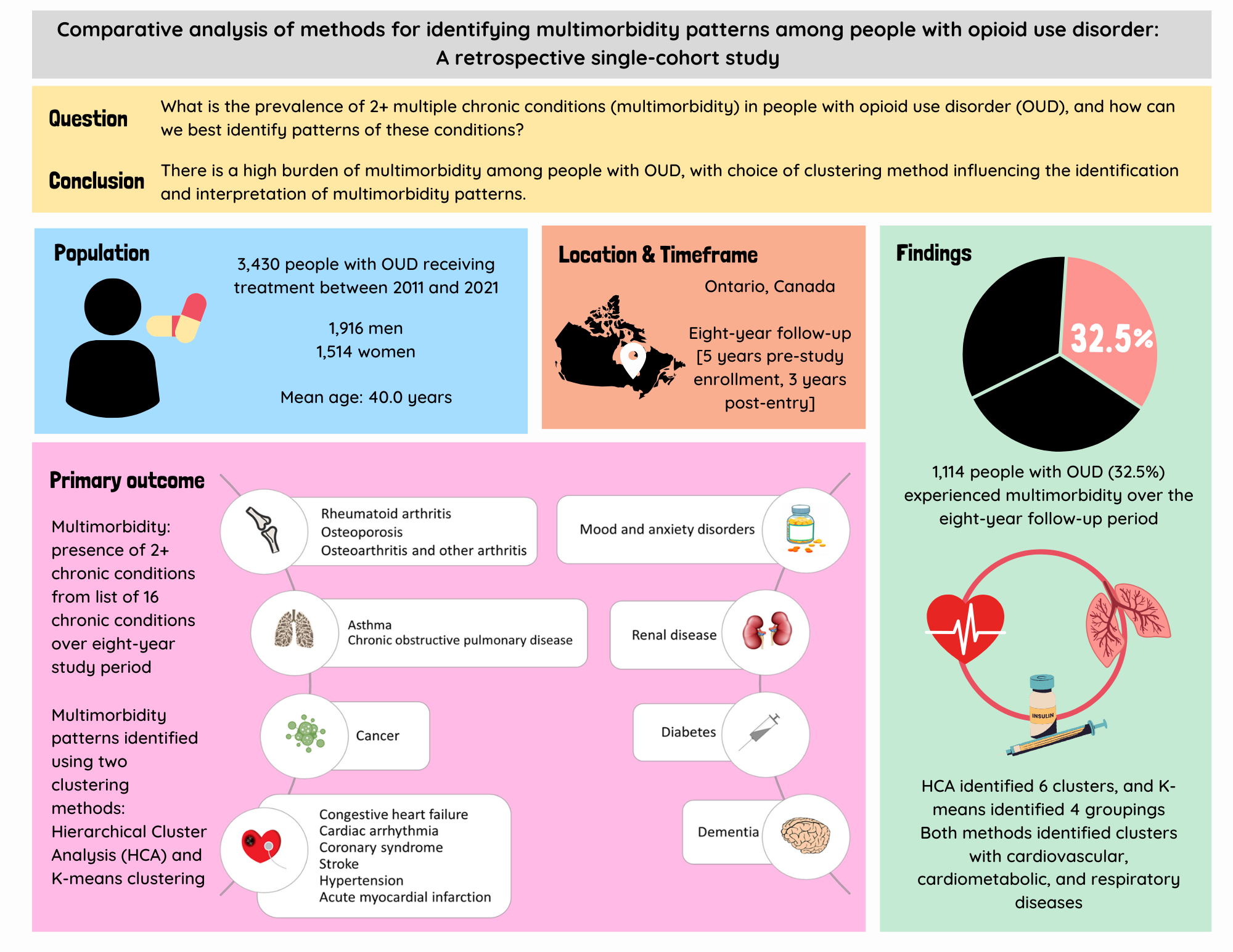

Supplement: S3 File — (TIF) [file pone.0324548.s003.tif]
